# Explaining residents' support to protect Gejia batik through the value-attitude-behavior model and theory of planned behavior

**DOI:** 10.1016/j.heliyon.2024.e30205

**Published:** 2024-04-25

**Authors:** Xizhen Li, Nurul Hanim Romainoor

**Affiliations:** a School of the Arts, Universiti Sains Malaysia, Penang, 11800, Malaysia; b School of Design and Art, Yancheng Institute of Technology, Yancheng, 224001, China

**Keywords:** Intangible cultural heritage, Gejia batik, VAB model, TPB theory, Residents' support

## Abstract

Much research focuses on customers' satisfaction with intangible cultural heritage products, with little consideration given to residents' support toward intangible cultural heritage. Therefore, this study focuses on constructing a conceptual framework that connects the theories of the value-attitude-behavior (VAB) model and planned behavior (TPB), aiming to explain residents' behavioral support for intangible cultural heritage (Gejia batik). To test the proposed model, we collected 412 sets of on-site survey data from four representative Gejia villages, namely Fengxiang, Wangba, Tangdu, and Matang. Six out of seven examined hypotheses were supported. The results showed that social value, aesthetic value, economic value, historical value, and perceived behavioral control of residents were positively and significantly related to residents' behavioral intention, which explained 46.3 % of the variance in behavioral intention. A positive and significant relationship exists between residents' attitudes and their behavioral intentions. Residents' attitude is an intermediary between social value, aesthetic value, economic value, historical value, perceived behavioral control, and residents' behavioral intention. This work provides theoretical and practical support for government departments in formulating protective policies related to Gejia batik.

## 1 Introduction

With the rapid development of modern society and the economy, the production methods of industrial civilization are gradually replacing those of agricultural civilization. In this context, traditional crafts in agricultural civilization have also been severely impacted. In 2003, the United Nations Educational, Scientific and Cultural Organization (UNESCO) officially incorporated the concept of intangible cultural heritage (ICH) into the international cultural heritage protection framework through the Convention for the Safeguarding of the Intangible Cultural Heritage. This convention aims to protect and promote ICH worldwide, including traditional performing arts, oral traditions and expressions, social practices, customs, rituals, festivals, handicrafts, and so on [1]. With the world's heightened attention to the protection of ICH, the emergence of ICH tourism models, and the advent of the experience economy era, tourists' travel preferences are no longer confined to traditional sightseeing. Instead, they are gradually evolving into deep, personalized, experiential tourism. In comparison to passive acceptance of pre-arranged tours, some tourists prefer to participate actively in the entire travel process [2]. Tourist destinations are now exploring their local ICH resources to meet the personalized needs of tourists. The experience of ICH and the purchase of ICH products have become gradually appealing aspects of cultural attraction for tourist destinations. These measures foster cultural prosperity in communities and enhance the sustainability of the local economy and society. Therefore, exploring and preserving ICH to achieve sustainable development has become a common goal for local stakeholders. Gejia batik is a traditional ICH craft practiced by ethnic minorities in the southeast Guizhou Province, China. It has been passed down through generations by diligent and kind Gejia women and was officially recognized as a national-level ICH in 2011 [3]. Gejia batik plays a special role in the local tourism industry. However, due to the complexity of handmade batik production, which involves multiple steps such as outlining, waxing, dyeing, dewaxing, cleaning, and drying, the process is time-consuming, and the profits are minimal. What's more, many residents work outside, far away from their hometown, to make a living. As a result, some capable individuals who could protect Gejia batik have sought employment elsewhere, and there is a growing trend of young people entering different industries, leading to a gap in the succession of Gejia batik craftsmanship. Su [4] believes that inheritors are the core of ICH. In the development of the ICH tourism industry, due to its intangible attribute, the transmission of ICH resources requires tangible individuals. The most suitable candidates for inheritance are the residents who genuinely understand the ICH [2].

In disseminating and protecting ICH, many researchers focus on the perspectives of consumers and tourists [[5], [6], [7]]. However, only limited attention is placed on the residents protecting the ICH. Given the gap mentioned above, this study attempts to integrate the Value-Attitude-Behavior (VAB) model with the Theory of Planned Behavior (TPB) to explain residents' support for the intention to protect Gejia batik. This research focuses on three main questions: What factors influence residents' attitudes and willingness to protect Gejia batik? How are these factors related to residents' willingness to protect batik? What role do residents' attitudes play in this context?

This study was the first time to combine the revised VAB model with TPB in the field of ICH protection. The use of complementary frameworks to explain residents' attitudes has received support from Ward and Berno [8] and has been substantiated in recent studies [9,10]. Understanding residents' perceived values of Gejia batik and combining this perception with the TPB contributes to understanding the drivers behind residents' engagement in ICH protection and enriches the theoretical framework for heritage preservation. Furthermore, this study provides viable strategies for local government to support and protect the ICH projects, thereby guiding the sustainable development of local heritage.

## 2 Literature review

### 2.1 Protecting ICH

Before the UNESCO Washington Conference in 1991, little attention was given to the inheritance and preservation of intangible heritage. The concept of “intangible heritage” was firstly introduced at Washington Conference by UNESCO. In 2003, the “Convention for the Safeguarding of Intangible Cultural Heritage” gave substantive policy to the global protection of ICH, strengthening the efforts of countries worldwide in exploring and safeguarding their ICH [11]. In the 21st century, an increasing number of scholars have joined the research on disseminating and protecting ICH. Categorically, some studies primarily focus on documenting craft methods. For instance, Shen [12] studied the application rules of stitching in different types of Beijing embroidery, the relationship between Beijing embroidery categories and stitching techniques, and supplemented and refined the research data of Beijing embroidery. He [13] conducted on-site investigations and research on the weaving of the Pu'er Wa ethnic group in Yunnan, exploring the preparation techniques of commonly used dyes in Wa brocade weaving. The study also investigated the looms and weaving techniques employed in the Wa brocade weaving. Some studies primarily focus on providing recommendations and suggestions. For example, Sun [14] explores how to activate positive factors to facilitate the successful inheritance of ICH from the perspective of knowledge transfer and incentive mechanisms, which concluded that cost control should be the first consideration in all economic measures. Chung [15] researched Hong Kong Cantonese opera, revealing the intricate relationship between technological usage and cultural dissemination. Emphasis was placed on preserving the inherent traditional core values, identity, and artistic aspects in the performance arts as part of ICH.

Regarding research objects, previous work mainly focused on the consumers. For example, Sun [16] investigated China's ICH, precisely the Huaihe River willow weaving technique. Initially, the study explored consumer purchasing intentions. Subsequently, four sustainable design improvement strategies for willow weaving product development were proposed using the analytic hierarchy process. Finally, considering the consumer perspective, the study revealed the sustainable impact of traditional craft design innovation in willow weaving on business and economic development. Zhang [7] took ICH products as a case study to explore the impact of consumer cultural identity and consumer knowledge on consumer purchasing intentions. The results indicate that cultural identity positively affects the intention to purchase ICH products, and the higher the consumer's knowledge level, the stronger this influence becomes. Some scholars focus on tourists as the research subjects. For example, Xiao [17] focused on the Changdao Fishermen's work song in China to assess tourists' preferences and willingness to pay for various conservation measures. The research indicates that tourists have a strong preference and a higher willingness to pay for the performance and preservation of the Fishermen's Songs of Changdao. Other scholars choose government institutions as the subjects of their research. For instance, Xu [18] documented and analyzed the legislative and policy efforts of the Chinese government in protecting ICH. The study indicates that China currently adopts a production-based paradigm for safeguarding ICH and is implementing some practice-oriented policy initiatives. Chinese legislative and policy endeavors in protecting ICH are impressive compared to most other countries. Based on the above analysis, few scholars have explored the protection and sustainable development of ICH from residents' perspective. Residents contribute more significantly to protecting ICH than tourists. Community involvement in preserving ICH can increase trust and public consensus, revealing inheritance strategies that meet local needs. Therefore, this study takes Gejia residents as the research objects.

### 2.2 VAB theory

Homer and Kahle [19] were the first to propose a VAB hierarchy model and apply it to the study of natural food consumption. The results suggest that values influence people's attitudes toward natural foods, and then influence people's purchase intention. Moreover, attitudes play a mediating role between values and behaviors. Value cognition is the foundation for guiding people's behavior and individuals' value judgment on something impacts his/her attitude toward it or the adoption of a particular behavior [4]. The value dimensions associated with the VAB hierarchy are divided into external and internal values. Although this value dimension has been widely applied to tourist destinations, ecological behavior, and other fields, the value of ICH cannot be solely described based on internal and external values. The “Convention for the Safeguarding of the Intangible Cultural Heritage” consistently emphasized the universal value of ICH [20]. The convention calls for societal attention to heritage's intangible values, such as historical, aesthetic, social, scientific, and other values. However, different individuals hold different perceptions of the value of ICH. Based on the available literature, the stakeholders protecting ICH include inheritors, governments, tourists, residents, tourism developers, and others. These diverse stakeholders have different perspectives on the value of ICH. For instance, the government places greater emphasis on the social and symbolic value of ICH [21]; the general public and tourists focus more on the practical and aesthetic value [22]; inheritors prioritize the spiritual value of ICH [23]; and tourism developers prioritize its economic value [24].

Throsby [25] and Hutter [26] argue that the value of ICH can be simply divided into cultural value and economic value. The economic value of ICH is almost universally recognized because it creates employment opportunities and compensates workers [27]. Regarding cultural value, Avrami [28] et al. suggest that cultural value includes historical, aesthetic, social, symbolic, and spiritual values. Ahmad [29] believes protecting cultural values encompasses aesthetic, social, scientific, and historical values. Xinhe [30] contends that the artistic value of ICH is an essential part of cultural value. Due to the limitations of human cognition, ICH also possesses many potential values. Because the research object of different studies is different, which leads to different value types.

**Fig. 1:**
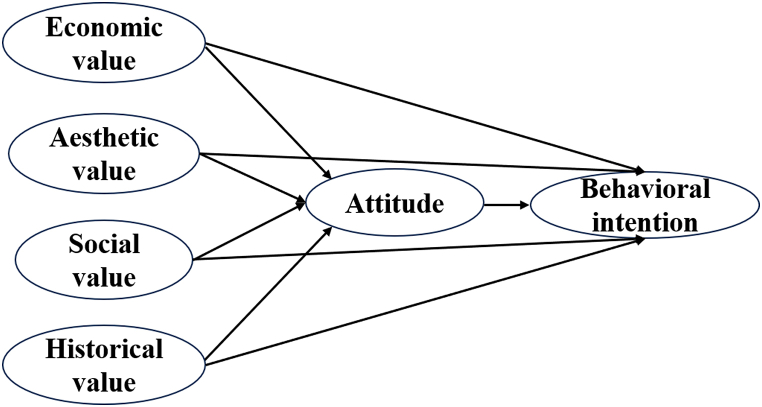
Revised theoretical framework of VAB model.

Qiu's [31] research confirms that residents' cognitive values of intangible cultural heritage (including social and economic value, educational and spiritual value, aesthetic value, and historical value) have a significantly positive relationship with residents' tourism intentions, and attitudes play an intermediate role between value cognition and tourism intentions. So, considering the findings of previous research [4,[32], [33], [34]] and the characteristics of batik, this work categorizes values into economic, aesthetic, social, and historical. Thus, following hypotheses are postulated, and the revised VAB model is illustrated in Fig. 1.

Hypothesis 1 (H1) Economic value is positively and significantly associated with residents' attitudes (H1a) and behavioral intention (H1b); residents' attitude mediates the relationship between economic value and behavioral intention (H1c).

Hypothesis 2 (H2) Aesthetic value is positively and significantly associated with residents' attitudes (H2a) and behavioral intention (H2b); residents' attitude mediates the relationship between aesthetic value and behavioral intention (H2c).

Hypothesis 3 (H3) Social value is positively and significantly associated with residents' attitudes (H3a) and behavioral intention (H3b); residents' attitude mediates the relationship between social value and behavioral intention (H3c).

Hypothesis 4 (H4) Historical value is positively and significantly associated with residents' attitudes (H4a) and behavioral intention (H4b); residents' attitude mediates the relationship between historical value and behavioral intention (H4c).

### 2.3 TPB theory

The TPB was proposed by the renowned social psychologist Icek Ajzen [35], which has been used in social sciences to explain human behavior for about forty years. It has evolved from the framework of the Theory of Reasoned Action (TRA) proposed by Fishbein and Ajzen to understand rational behavior. The TRA posits that personal rationality will entirely control individual behavior [36]. However, numerous empirical findings suggest that the TRA often fails to provide a reasonable explanation for behaviors that are not entirely within individual volitional control. Therefore, Ajzen modified and developed the TPB based on the TRA to address these limitations [35]. The traditional TPB consists of five factors: attitudes, subjective norms, perceived behavioral control, behavioral intentions, and behavior [35]. The predictive performance of the TPB on behavior is as follows: attitudes, subjective norms, and perceived behavioral control have a positive and significant impact on behavioral intentions; behavioral intentions have a positive and significant impact on actual behavior, and perceived behavioral control plays a moderating role between behavioral intention and actual behavior.

Because the TPB provides an explanatory framework for individual behavioral intentions and actual behavior, demonstrating predictive solid power, it has been widely applied to explain and predict various behaviors, including but not limited to the fields of health sciences, environmental sciences, consumer behavior, tourism, education, and more. For instance, Liu [37] applied the TPB theory to the field of English education to measure the behavioral intention of language teachers to incorporate informal digital learning of English into formal curriculum context. The research results indicate that subjective norms, perceived behavioral control, and attitudes all have a positive and significant relationship with behavioral intention. Hasan [38] used the extended TPB theory to examine consumers' purchase intentions towards green tea and found that attitude and perceived behavioral control both have a positive and significant impact on green tea purchase intentions. In recent years, applying the TPB in tourism and ICH protection has also become increasingly active. Xia [39] developed an extended TPB model to examine the effectiveness of public participation in the intentions and behaviors of ICH inheritance from the perspective of multi-subject collaboration. The results indicate that the expanded behavioral theory can be used to evaluate the collaborative processes in inheriting ICH. Wu [40] took college students as the research objective and developed an extended TPB model to explore the factors influencing college students' travel intentions in cultural heritage tourism. The results show that college students' generative power, attitude towards cultural heritage tourism, subjective norms, and perceived behavioral control are significantly related to their intention to participate in cultural heritage tourism. Hsu [41] discussed the sustainable development of ICH tourism from the perspective of a rational behavior process based on the TPB, which illustrate that not only altruistic intention but also egoistic benefit has a positive and significant relationship with ICH tourism decision-making.

**Fig. 2:**
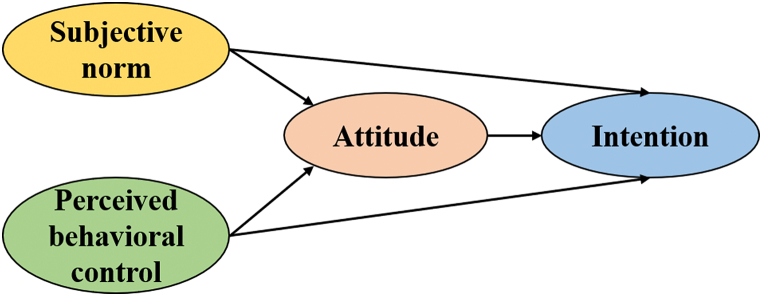
Revised theoretical framework of Planned behavior.

In the earlier framework of the TPB, attitudes, subjective norms, and perceived behavioral control were considered parallel variables jointly influencing behavioral intentions, with no interaction between them. However, later empirical studies revealed that subjective norms and perceived behavioral control indirectly affect behavioral intentions through attitudes [[42], [43], [44]]. As such, this study proposed the following hypothesis, as shown in Fig. 2.

Hypothesis 5 (H5) Subjective norm is positively and significantly associated with residents' attitudes (H5a) and behavioral intention (H5b); residents' attitude mediates the relationship between subjective norm and behavioral intention (H5c).

Hypothesis 6 (H6) Perceived behavioral control is positively and significantly associated with residents' attitudes (H6a) and behavioral intention (H6b); residents' attitude mediates the relationship between perceived behavioral control and behavioral intention (H6c).

Hypothesis 7 (H7) Residents' attitude is positively and significantly associated with behavioral intention

**Fig. 3:**
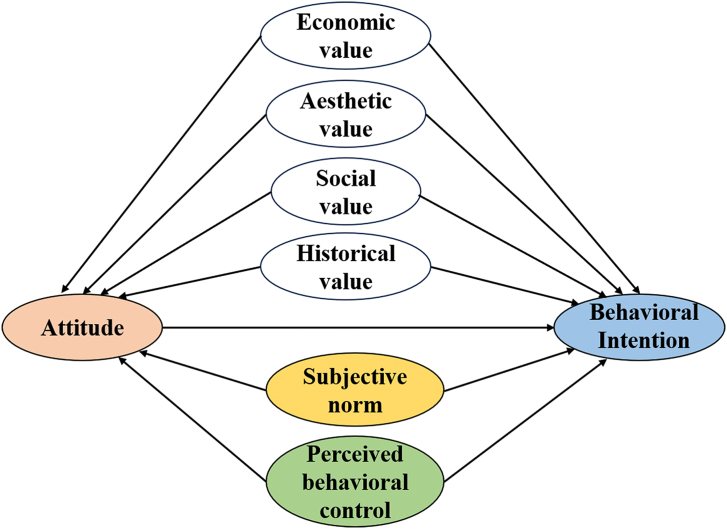
The theoretical framework being tested.

Based on the literature review and the construction of revised VAB and TPB model, we assume that value cognition includes economic value, social value, aesthetic value, and historical value, all of which have a positively significant relationship with residents' attitudes and behavioral intentions. At the same time, subjective norms and perceived behavioral control also have a positively significant relationship with residents' attitudes and behavioral intentions. Attitudes play an intermediary role between these factors and residents' behavioral intentions. The assumptions were formulated as Fig. 3.

## 3 Research methodology

### 3.1 Participants

In this study, purposive sampling will be used to select the participants. The selection of participants should meet the inclusion criteria and exclusion criteria shown in the following to offer relevant information regarding this research.

#### 3.1.1 Inclusion criteria

First, the participants in the study were those who identified as being of Gejia descent. Second, participants must have lived in one of the four villages for more than 10 years. This is crucial because the participants had to be acquainted with the study region since the study focuses on residents' intentions to engage with their Batik. Third, participants must be at least 18 years old. This is important because adults over 18 are better able to understand the purpose, risks, and benefits of their participation in research and can make independent decisions about whether to participate.

#### 3.1.2 Exclusion criteria

First, participants who need help understanding or communicating effectively in Mandarin will be excluded. Second, people with physical disabilities, such as deafness or blindness were not included in the study. Because the researchers don't have the resources or knowledge to provide necessary accommodations for individuals with disabilities to participate fully in the study. Third, Participants who are unwilling to provide informed consent will be excluded.

### 3.2 Instrument development

This study adopts the paradigm of positivist and conducts research by designing a quantitative survey questionnaire to collect data [45]. The questionnaire consists of two parts. The first part is mainly demographic information, including gender, age, ethnicity, marital status, education level, monthly income, village of residence, and length of residence. The second part consists of the main consturcts of the VAB and TPB models, including economic value, aesthetic value, social value, historical attitude, subjective norm, perceived behavioral control, attitude, and behavioral intention. The four constructs of aesthetic, social, economic, and historical value were adapted from Sun's [4] and Qiu's [31] studies. The remaining four constructs, subjective norm, perceived behavioral control, attitude, and behavioral intention, are adapted from Erul's [46] and Luo's [11] research. We invited three experts in this research field to check the content validity of the questionnaire, including whether each question is appropriate, whether the questions in each dimension can truly represent this dimension, and whether the questions are easy to understand by the respondents. Based on expert feedback, we have removed and revised some questions. Finally, there are 33 questions left, as shown in Table 1. The seven-point Likert scale was used for all variables to measure the respondents' attitudes or opinions. The Likert seven-point scale provides more levels, allowing participants to have more choices in expressing their attitudes. Each respondent can choose a score of 1–7(1 = strongly disagree; 2 = disagree; 3 = somewhat disagree; 4 = neutral; 5 = somewhat agree; 6 = agree; 7 = strongly agree) based on their feelings and experience.

**Table 1:** Statements of the questionnaire content.

Constructs	Statements	Sources
Economic value (EV)	**EV1:** I think the commercial development of Gejia Batik can benefit the inheritor.	[4]
	**EV2:** I think developing Gejia Batik can generate economic income for the local area.	
	**EV3:** I think the protection of Gejia Batik can promote the employment of local people.	
	**EV4:** I think Gejia Batik has economic value.	
Aesthetic value (AV)	**AV1:** I think Gejia Batik can give people aesthetic enjoyment.	[4,31]
	**AV2:** I think Gejia Batik aligns with contemporary people's aesthetics.	
	**AV3:** I think Gejia Batik has artistic appeal.	
	**AV4:** I think Gejia Batik has unique characteristics and styles.	
Social value (SV)	**SV1:** I think Gejia Batik reflects cultural diversity.	[4]
	**SV2:** I think Gejia Batik has a cultural influence.	
	**SV3:** I think Gejia Batik is an essential achievement of human civilization.	
	**SV4:** I think Gejia Batik can enhance local influence.	
Historical value (HV)	**HV1:** Gejia Batik has a long history.	[4]
	**HV2:** Gejia Batik supplements the deficiency of traditional historical records.	
	**HV3:** Gejia Batik is a reflection of and witness to historical development.	
	**HV4:** Gejia Batik is a reflection of the cultural tradition of the community.	
	**HV5:** Gejia Batik continues history and culture in the contemporary era.	
Subjective norm (SN)	**SN1:** People whose opinions I value would prefer that I support Gejia Batik's development.	[11,46]
	**SN2:** People who are important to me think I should support Gejia Batik's development.	
	**SN3:** People who are vital to me suggested I protect Gejia Batik.	
	**SN4:** My friends encourage me to participate in the protection of Gejia Batik.	
Perceived behavioral control (PBC)	**PBC1:** I have the skills to perform work to support the development of Gejia Batik.	[46]
	**PBC2:** I have the talent to perform work to support the development of Gejia Batik.	
	**PBC3:** I can perform work to support the development of Gejia Batik.	
	**PBC4:** I have time to support Gejia Batik's development.	
Attitude (ATT)	**ATT1:** I am very interested in protecting Gejia Batik	[11]
	**ATT2:** I think Gejia Batik is worth protecting.	
	**ATT3:** I think it is essential to protect Gejia Batik	
	**ATT4:** I have the responsibility to protect Gejia batik.	
Behavioral intention (BI)	**BI1:** I will make an effort to support the development of Gejia Batik.	[46]
	**BI2:** I plan to support the development of Gejia Batik	
	**BI3:** I am willing to support the development of Gejia Batik.	
	**BI4:** I want to work in the conservation of Gejia batik.	

### 3.3 Sampling and data collection

This study uses a cross-sectional data collection method, so the data is collected all at once [45]. The target population is the Gejia people residing in Southeast of Guizhou. Researchers selected four representative Gejia villages as the main research sites. The Gejia people from these four villages form the sampling frame for our study. The participants must have lived in one of the four villages for more than 10 years. The sampling method used in this study is purposive sampling.

The data collection is conducted in two phases. The first round of data collection is for pilot data. In order to ensure the representativeness of the sample, we conducted a pilot survey in four villages, selecting individuals of different genders, ages, educational backgrounds, and monthly incomes. Seventy-two samples were collected, meeting the specified requirements for the pilot data quantity as suggested by the experts [47]. The second round of data collection is for the formal data collection. The sample size for formal data collection is based on the research of Krejcie and Morgan [48]; they recommended a formula (as Eq. (1)) to calculate the sample size (s) based on the total population (N). Since the total population of Gejia people is approximately 50,000, according to this formula, the minimum sample size required for this study is calculated to be 385. Therefore, 412 valid samples were collected in this study, which met the sample size requirements. In addition, the sample size for each village is calculated according to the total sample size and the proportions of Gejia population in the four villages, as shown in Table 2. The formal data collection lasted nearly two months, from August 15, 2023, to October 15, 2023. The survey was conducted through on-site investigations, collecting data through purposive sampling in Fengxiang, Wangba, Tangdu, and Matang—— the four densely populated villages inhabited by the Gejia people.

(1) $$s=\frac{X^{2}NP\left(1-P\right)}{d^{2}\left(N-1\right)}+X^{2}P\left(1-P\right)$$

**Table 2:** Sample size for each village.

Village	Total population	sample size
Tangdu	2000	412×[2000÷(2000+3700+450+1400)]≈109
Fengxiang	3700	412×[3700÷(2000+3700+450+1400)]≈202
Matang	450	412×[450÷(2000+3700+450+1400)]≈25
Wangba	1400	412×[1400÷(2000+3700+450+1400)]≈76

s = required sample size.

X^2^ = the table value of chi-square for 1 degree of freedom at the desired confidence level (3.841).

N = the population size.

P = the population proportion (assumed to be 0.50 since this mold provide the maximum sample size).

d = the degree of accuracy expressed as a proportion (0.05).

## 4 Results

### 4.1 Item analysis

We conducted item analysis on the pilot data to examine whether our proposed scale had discriminative power for the participants. The pilot data sample comprised 72 Gejia people from different villages (including Fengxiang, Wangba, Tandu, and Matang villages), ages, and social backgrounds. SPSS v.26 is used to analyze the pilot data. Firstly, we aggregate the distribution of all items within each dimension. Subsequently, we identified the values corresponding to the 27th and 73rd percentiles and used them as standards to categorize the data into low and high groups. Finally, independent t-tests were conducted for each item within each dimension between the two groups. If there was a significant difference in means, it indicated that the item had discriminative power; otherwise, it should be considered for removal. The results of the *t*-test showed that except for SV4, AV4, and SN4, there were significant differences in the mean values between the high and low groups of all variable items (p＜0.050) (Appendix Table A). Therefore, the finalized questionnaire, comprising 30 items, is well-suited for a formal investigation.

### 4.2 Common method variance

Common method variance is a problem that behavioral science researchers need to pay attention to [49]. Many researchers believe that measuring two or more structures in the same way may have a biased effect on estimates of the relationship between them. This study employed several procedural control measures during questionnaire design and data collection to mitigate the impact of common method variance on research outcomes. Firstly, the meaning measured by the constructs was deliberately veiled during questionnaire design. Secondly, item content was articulated in a concise, easily understandable, and unambiguous manner. Thirdly, items from each dimension were arranged randomly. Lastly, during data collection, participants were explicitly informed about the survey's anonymity to encourage more candid and truthful responses. While this study implemented four procedural control measures to alleviate common method variance, the concentrated survey timeline, consistent questionnaire structure, consistency of survey methods, and the similarity of respondents' living environments unavoidably introduce the potential influence of common method bias on the collected data. Consequently, it is crucial to assess the extent of the impact of common method variance on the data. We adopted Harman's single-factor test to assess common method variance. We conducted an exploratory factor analysis with all variables, utilizing principal component analysis to extract factors with eigenvalues greater than 1, examining the unrotated factor analysis results. Subsequently, we assessed the explanatory power of the first factor. If the explanatory power of the first factor exceeds 50 %, it indicates a significant presence of common method variance. If it did not surpass 50 %, it suggests that common method bias was within an acceptable range [50]. In this work, the explanatory power of the first factor is 34.64 %. Therefore, common method variance is not a severe issue in this study.

### 4.3 Formal data processing and analysis

In this research, SPSS v.26 and Process 2.15 was employed to analysis the formal data. We conducted the formal investigation using purposive sampling. We selected the residents of Gejia who are willing to participate in the study and live in one of the four villages as the subjects of investigation. During the survey, 450 questionnaires were sent out; however, 38 questionnaires were invalid, in which 17 had missing values, and 21 chose the same option. After excluding invalid questionnaires, a total of 412 valid questionnaires were obtained, with a response rate of 91.6 %. The efficiency of the collected questionnaires reached the standard of statistical analysis.

#### 4.3.1 Demographic and statistical analysis of respondents

Through descriptive statistical analysis of the demographic characteristics of the sample data (Table 3), we found that there were 199 males, accounting for 48.3 % of the total sample size, and 213 females, accounting for 51.7 %. All the respondents were Gejia people. According to the data, young and middle-aged individuals constituted the majority, with 141 people aged 18–29 making up 34.2 %, 137 people aged 30–39 accounting for 33.3 %, and 134 people aged 40–60 representing 32.5 %. Regarding marital status, the percentage of married individuals was significantly higher at 73.3 % compared to unmarried individuals at 26.7 %. Regarding educational attainment, those with a high school education and below comprised 51.5 %, college graduates comprised 18.7 %, undergraduates accounted for 25.2 %, and postgraduates represented 4.6 %. Concerning monthly income, individuals earning less than 1000 yuan accounted for 26.7%, those earning 1000–3000 yuan accounted for 27.7 %, those earning 3001–5000 yuan comprised 22.3 %, those earning 5001–8000 yuan represented 16.5 %, and those earning 8001–10000 yuan constituted 6.8 %. Regarding sample distribution, those from Fengxiang Village accounted for 49 %, those from Wangba Village accounted for 18.4 %, those from Tangdu Village accounted for 26.5 %, and those from Matang Village accounted for 6.1 %. 59 % lived for 10–20 years, 24 % for 20–30 years, and 17 % for more than 30 years.

**Table 3:** Respondents’ demographic profile.

Item	Demographics	Frequency	Percent(%)	Item	Demographics	Frequency	Percent(%)
Gender	Male	199	48.3	Marriage	Married	302	73.3
	Female	213	51.7		Unmarried	110	26.7
Ethnic	Gejia	412	100.0	Monthly income(¥)	＜1000	110	26.7
Age	18–29	141	34.2		1000–3000	114	27.7
	30–39	137	33.3		3001–5000	92	22.3
	40–49	70	17.0		5001–8000	68	16.5
	50–60	64	15.5		8001–10000	28	6.8
Educational level	High school or below	212	51.5	Village	Fengxiang	202	49.0
	College	77	18.7				
	University	104	25.2		Wangba	76	18.4
	Postgraduate	19	4.6				
Live years	10–20	243	59.0		Tangdu	109	26.5
	20–30	99	24.0				
	＞30	70	17.0		Matang	25	6.1

#### 4.3.2 Factor analysis

To prove that the items of the scale have an underlying structure or dimension, we conducted a factor analysis. We first performed the Kaiser-Meyer-Olkin (KMO) and Bartlett's Test of Sphericity to prove that our data was suitable for factor analysis. The KMO measure should be greater than 0.7 and is inadequate if less than 0.5. The KMO value tells us whether or not enough items are predicted by each factor. Then, the Bartlett test should be significant(P < 0.05), which means that the variables are correlated highly enough to provide a reasonable basis for factor analysis. In this study, the KMO value was 0.911 > 0.7, and the P value of the Bartlett test was 0.000 (P < 0.05), which proved that our data were suitable for factor analysis. Then, principal axis factor analysis with varimax rotation was conducted to assess the underlying structure for the 30 questionnaire items. Finally, we extracted eight factors with eigenvalues larger than 1 from 30 items: social value, aesthetic value, economic value, historical value, subjective norm, perceived behavioral control, attitude, and behavioral intention. The overall explanatory power of the eight factors reaches 70.89 %, meeting the ideal criterion. Based on the criteria of factor analysis, we removed items with factor loadings below 0.6 and items that loaded onto incorrect factors. In the end, we obtained 29 items, as shown in Table 4.

**Table 4:** Rotated factor loading matrix.

	1	2	3	4	5	6	7	8
BI1	0.655							
BI2	0.734							
BI3	0.735							
BI4	0.751							
HV1		0.691						
HV2		0.687						
HV3		0.716						
HV4		0.731						
HV5		0.764						
SV1			0.839					
SV2			0.731					
SV3			0.756					
AV1				0.742				
AV2				0.811				
AV3				0.825				
EV1					0.734			
EV2					0.788			
EV3					0.782			
EV4					0.825			
PBC1						0.829		
PBC2						0.850		
PBC3						0.843		
SN1							0.794	
SN2							0.805	
SN3							0.744	
ATT1								0.783
ATT2								0.832
ATT3								0.823
ATT4								0.731

Extraction Method: Principal Component Analysis.

Rotation Method: Varimax with Kaiser Normalization.

Note: EV= Economic value; AV= Aesthetic value; SV=Social value; HV=Historical value; SN=Subjective norm; PBC=Perceived behavioral control; ATT = Attitude; BI=Behavioral intention.

#### 4.3.3 Reliability and validity

We conduct reliability and validity analyses on the questionnaire scales. Cronbach's alpha were computed to determine the consistency in measuring the same concept across all items in each scale. alpha exceeds 0.7 means that the result is reliable. An excessively high alpha (e.g., exceeding 0.90) indicates that the items are repetitious or you have more items in the scale than required for an internally reliable measure of the concept. In this work, the alpha is larger than 0.7 and lower than 0.9 for all scales, indicating that these items would collectively constitute eight scales with strong internal consistency reliability. Validity can be divided into convergent validity and discriminant validity. Convergent validity is used to assess the degree of consistency among different items (or observations) in measuring the same concept. The calculation results (as Table 5) show that the convergent validity of all constructs is greater than 0.5, indicating that the questionnaire has good convergent validity. Discriminative validity ensures that the construct is unique, and is not overlaps with other concepts. We employed Fornell-Lacker's criterion to calculate the discriminant validity of each construct. The results (as Table 5) show that the correlation of each construct with itself is higher than that with other constructs, suggesting that the questionnaire possesses good discriminant validity. Therefore, our questionnaire demonstrates sufficient reliability and validity, making it suitable for statistical analysis.

**Table 5:** Reliability and validity.

Construct	Convergent Validity		Mean	Std.Deviation	Discriminant Validity							
	Cronbach's Alpha	AVE			HV	SV	AV	EV	PBC	SN	ATT	BI
HV	0.839	0.516	4.94	0.998	**0.718**							
SV	0.822	0.616	4.89	1.013	0.406	**0.785**						
AV	0.822	0.632	4.84	0.995	0.374	0.340	**0.795**					
EV	0.881	0.622	4.29	1.149	0.424	0.337	0.319	**0.789**				
PBC	0.898	0.706	3.69	1.383	0.341	0.208	0.396	0.387	**0.840**			
SN	0.795	0.614	4.57	0.880	0.345	0.307	0.354	0.437	0.363	**0.784**		
ATT	0.896	0.628	5.18	1.032	0.468	0.391	0.415	0.403	0.366	0.327	**0.792**	
BI	0.858	0.517	4.58	1.054	0.490	0.424	0.443	0.499	0.487	0.408	0.549	**0.719**

Note: AVE = Average Variance Extracted; Std. Deviation = Standard Deviation; EV = Economic value; AV = Aesthetic value; SV=Social value; HV=Historical value; SN=Subjective norm; PBC=Perceived behavioral control; ATT = Attitude; BI=Behavioral intention.

#### 4.3.4 Regression analysis

We conducted a multiple regression analysis on the dependent variable and independent variables. Initially, we examined the relationships among social value, aesthetic value, economic value, historical value, subjective norm, and perceived behavioral control with attitudes. The results (as Table 6) indicate that, except for subjective norm, all other variables, including social value, aesthetic value, economic value, historical value, and perceived behavioral control, exhibited a positive and significant relationship with attitudes (P＜0.05). Additionally, the variance inflation factor was less than 5 (VIF＜5), indicating no multicollinearity issues among the independent variables. The R-squared for the impact of the six variables on attitudes is 0.351, indicating that these six variables collectively account for 35.1 % of the variance in attitude, reaching a moderate explanatory power level. Then, we conducted a linear regression on attitudes and behavioral intentions. The results revealed that residents' attitudes have a positive and significant relationship with behavioral intentions. What's more, the variance inflation factor was less than 5 (VIF＜5), indicating no multicollinearity issues among the independent variables. The R-squared for the impact of the attitude on behavioral intention is 0.302, indicating that attitudes explain 30.2 % of the variance in behavioral intention, reaching a moderate level of explanatory power. Finally, we examined the relationships among social value, aesthetic value, economic value, historical value, subjective norm, and perceived behavioral control with behavioral intention. The results indicated that, except for subjective norm, all other variables, including social value, aesthetic value, economic value, historical value, and perceived behavioral control, exhibited a positive and significant relationship with behavioral intention (P＜0.05). Additionally, the variance inflation factor was less than 5 (VIF＜5), indicating no multicollinearity issues among the independent variables. The R-squared for the impact of the six variables on behavioral intention is 0.463, indicating that these six variables collectively account for 46.3 % of the variance in behavioral intention.

**Table 6:** Regression analysis.

DV	IV	Unstandardized Coefficients		Standardized Coefficients	t	Sig.	Collinearity Statistics		R2
		B	Std. Error	Beta			Tolerance	VIF	
ATT	(Constant)	1.315	0.290		4.528	0.000			0.351
	HV	0.237	0.050	0.229	4.743	0.000	0.689	1.452	
	SV	0.161	0.047	0.158	3.451	0.001	0.763	1.310	
	AV	0.178	0.049	0.171	3.645	0.000	0.725	1.379	
	EV	0.122	0.044	0.135	2.784	0.006	0.678	1.475	
	PBC	0.091	0.035	0.122	2.604	0.010	0.734	1.363	
	SN	0.042	0.055	0.035	0.751	0.453	0.718	1.392	
BI	(Constant)	1.670	0.223		7.502	0.000			0.302
	ATT	0.561	0.042	0.549	13.314	0.000	1.000	1.000	
BI	(Constant)	0.237	0.270		0.879	0.380			0.463
	HV	0.192	0.046	0.182	4.152	0.000	0.689	1.452	
	SV	0.171	0.043	0.165	3.953	0.000	0.763	1.310	
	AV	0.145	0.045	0.137	3.209	0.001	0.725	1.379	
	EV	0.184	0.041	0.201	4.541	0.000	0.678	1.475	
	PBC	0.177	0.032	0.232	5.464	0.000	0.734	1.363	
	SN	0.088	0.051	0.074	1.718	0.087	0.718	1.392	

Note: DV = Dependent Variable; IV=Independent Variable; Std. Deviation = Standard Deviation; EV = Economic value; AV = Aesthetic value; SV=Social value; HV=Historical value; SN=Subjective norm; PBC=Perceived behavioral control; ATT = Attitude; BI=Behaviral intention; R^2^=R square; VIF=Variance Inflation Factor; Sig. = Significance; t = t-value.

#### 4.3.5 Exploring the mediating role of attitude

We test the mediating relationship in this study use SPSS v.26 plus Process 2.15. We used a basic mediator model with multiple predictors to investigate the indirect associations within the model (model 4, sampling 5000 times). The analysis results are shown in Table 7. We conducted a statistical mediation analysis to determine if attitude mediates the relationship between independent variables and behavioral intention. For the indirect relationship of aesthetics value (economic value, historical value, social value, subject norm, and perceived behavioral control) on behavioral intention, the beta for this effect is 0.043 (0.029, 0.057, 0.039, 0.010 and 0.022), with a bootstrapped standard error (BootSE) of 0.018 (0.015, 0.019, 0.020, 0.024, and 0.011) and a 95 % confidence interval (BootLLCI and BootULCCI) ranging from 0.015 (0.006, 0.026, 0.011, −0.022, and 0.006) to 0.091 (0.066, 0.103, 0.094, 0.079, and 0.053). It means that attitude statistically significantly mediates the relationship between other factors and behavioral intention if zero does not located in the 95 % confidence interval. Hence, attitude statistically significantly mediates the relationship between behavioral intention and all constructs (including aesthetics value, economic value, historical value, social value, and perceived behavioral control) except subject norms.

**Table 7:** Mediating-effect analysis.

Path	Effect	Std.E	LLCI	ULCI
Indirect effect: AV→ATT→BI	0.043	0.018	0.015	0.091
Indirect effect: EV→ATT→BI	0.029	0.015	0.006	0.066
Indirect effect: HV→ATT→BI	0.057	0.019	0.026	0.103
Indirect effect: SV→ATT→BI	0.039	0.020	0.011	0.094
Indirect effect: SN→ATT→BI	0.010	0.024	−0.022	0.079
Indirect effect: PBC→ATT→BI	0.022	0.011	0.006	0.053

Note: Std. E = Standard Error; LLCI = Lower Level Confidence Interval; ULCI=Upper Level Confidence Interval; EV = Economic value; AV = Aesthetic value; SV=Social value; HV=Historical value; SN=Subjective norm; PBC=Perceived behavioral control; ATT = Attitude; BI=Behavioral intention.

#### 4.3.6 Hypothesis-testing results

We have examined all hypotheses through multiple regression analysis and intermediary effect analysis of variables in the model. The critical information was extracted through Table 5, Table 6, Table 7, and the summary is presented in Table 8.

**Table 8:** Hypothesis-testing results.

Hypotheses	Sub-hypotheses	Path	Std.SE	P	Result
H1	H1a	Economic value is positively and significantly associated with residents' attitudes.	0.044	0.006	Supported
	H1b	Economic value is positively and significantly associated with residents' behavioral intention.	0.041	0.000	Supported
	H1c	Residents' attitude mediates the relationship between economic value and behavioral intention.	0.012	0.015	Supported
H2	H2a	Aesthetic value is positively and significantly associated with residents' attitudes.	0.049	0.000	Supported
	H2b	Aesthetic value is positively and significantly associated with residents' behavioral intention.	0.045	0.001	Supported
	H2c	Residents' attitude mediates the relationship between aesthetic value and behavioral intention.	0.014	0.003	Supported
H3	H3a	Social value is positively and significantly associated with residents' attitudes.	0.047	0.001	Supported
	H3b	Social value is positively and significantly associated with residents' behavioral intention	0.043	0.000	Supported
	H3c	Residents' attitude mediates the relationship between social value and behavioral intention	0.014	0.004	Supported
H4	H3a	Historical value is positively and significantly associated with residents' attitudes.	0.050	0.000	Supported
	H3b	Historical value is positively and significantly associated with residents' behavioral intention	0.046	0.000	Supported
	H3c	Residents' attitude mediates the relationship between historical value and behavioral intention.	0.016	0.000	Supported
H5	H5a	Subjective norm is positively and significantly associated with residents' attitudes.	0.055	0.453	Reject
	H5b	Subjective norm is positively and significantly associated with residents' behavioral intention	0.051	0.087	Reject
	H5c	Residents' attitude mediates the relationship between subjective norm and behavioral intention.	0.014	0.465	Reject
H6	H6a	Perceived behavioral control is positively and significantly associated with residents' attitudes.	0.035	0.010	Supported
	H6b	Perceived behavioral control is positively and significantly associated with residents' behavioral intention.	0.032	0.000	Supported
	H6c	Residents' attitude mediates the relationship between perceived behavioral control and behavioral intention.	0.009	0.021	Supported
H7	H7	Residents' attitude is positively and significantly associated with behavioral intention.	0.042	0.000	Supported

Note: P=P-value; Std. SE=Standard Error of the Estimate; H1=Hypothesis1; H2=Hypothesis2; H3=Hypothesis3; H4=Hypothesis4; H5=Hypothesis5; H6=Hypothesis6; H7=Hypothesis7.

Based on the results of hypothesis tested, as shown in Table 7, we can infer that H1, H2, H3, H4, H6, and H7 are all supported except for H5. Therefore, we can conclude that economic value, aesthetic value, social value, historical value, and perceived behavioral control have a positive and significant relationship with residents' attitudes and behavioral intention. What's more, residents' attitudes play a mediating role between economic value, aesthetic value, social value, historical value, perceived behavioral control, and residents' behavioral intention. Although the subjective norm positively relates to residents' attitudes and behavioral intentions, the relationship is non-significant.

## 5 Discussion and conclusion

Previous literature claims that modifying planned behavior models by adding key factors or changing paths can increase our understanding of behavior and predict a person's behavioral intention [46]. Therefore, in this study, we attempt to reconstruct a new theoretical model based on the VAB model and the TPB model to explain the conservation intention of residents towards Gejia batik. The dependent variable in the model is behavioral intention, and the independent variables include economic value, aesthetic value, social value, historical value, subjective norm, and perceived behavioral control. Attitude serves as the mediating variable.

This study found that economic value has a positive and significant relationship with residents' attitudes and behavioral intentions. This is similar to Vijaranakorn's [51] research findings in the luxury products, where economic value and purchasing intention are positively correlated. This finding is also consistent with Qiu's work [31], in which the economic value of ICH is crucial in garnering residents' support for its development. ICH projects typically possess economic potential and can become integral components of tourism, handicrafts, or cultural and creative industries. The residents will present more interest in conserving ICH if they recognize that preserving ICH contributes to job creation and income enhancement and promotes sustainable development. Furthermore, aesthetic value has a positive and significant relationship with residents' attitudes and behavioral intention. This research finding is consistent with Li's [52] research on consumer's purchase intention toward cultural and creative products and Yu's [53] study on consumers' willingness to purchase Upcycled Products. We know that ICH is typically manifested through tangible product. When residents realize that ICH products possess unique aesthetic value, they may feel a sense of cultural identity and pride, leading more willing to actively participate in and support the conservation of ICH.

Historical value also has a positive and significant relationship with residents' attitudes and behavioral intentions, supporting Qiu's [31] research results. ICH typically carries the sedimentation of history, reflecting lifestyles, values, and skills from past eras. Residents may perceive the historical value of ICH as part of their cultural heritage, believing that by preserving these traditions, they can maintain and pass on their roots and cultural identity. Moreover, social value has a positive and significant relationship with residents' attitudes and behavioral intentions. This finding supports the results of Chen [54] and Qiu's [31] research. ICH represents cultural diversity, and the conservation of ICH contributes to maintaining cultural pluralism. Additionally, as a shared wealth of society, ICH plays a role in uniting community members. By preserving ICH, residents may feel they collectively share a unique cultural heritage, enhancing their sense of social identity and cohesion. Therefore, enhancing residents' awareness of the value of ICH is crucial for its preservation. The decision-makers of the government departments should educate the residents by combining the economic, historical, aesthetic, and social values of the ICH so that residents can realize the importance of Gejia batik to the region and the nation from the heart.

Based on the results of this study, perceived behavioral control has a significant positive relationship with residents' attitudes and behavioral intentions, supporting the findings of Liu's [37] and Wu's [40] research. Moreover, from the standpoint of standardized coefficients, the coefficient for perceived behavioral control is the highest, indicating its utmost importance for behavioral intentions. Perceived behavioral control refers to an individual's subjective perception of the degree of control over their behavior, including their confidence in the ease or difficulty of the behavior and their capabilities. When residents in the study area have mastered the techniques of batik production and possess the necessary equipment, facilities, time, funds, and other conditions to protect traditional batik, they are more likely to exhibit a positive intention to preserve it, demonstrating a willingness to invest additional effort and resources.

However, the results show that although subjective norms have a positive relationship with residents' attitudes and behavioral intentions, they are insignificant. This finding supports Shen's [55] research but is inconsistent with most scholars' findings [56,57]. Although our research expected that subjective norms would play a positive role in protecting Gejia batik, empirical results showed that subjective norms did not significantly relate to residents' attitudes and behavioral intentions. This result provides some insights worth exploring further. First, there may be a low degree of social expectation and consensus on batik conservation in the community. Social expectations usually form part of subjective norms. However, the role of subjective norms may be weakened if there is a low consensus among community residents on the cultural importance and conservation value of batik. Second, individual independence and autonomy have played an essential role in this community. Residents may emphasize individual independent thinking and autonomous behavior, with relatively little influence on social expectations and the views of others. This independence make the influence of subjective norms relatively weak since individuals are more inclined to formulate behavioral intentions based on their values and judgments.

The residents' attitudes have a positive and significant relationship with their behavioral intentions, consistent with findings in various fields [31,39,58]. The correlation between residents' attitudes and behavioral intentions is well-established, indicating that the more positive the attitude, the stronger the residents' intention to engage in a particular behavior. Kraus [59] points out that attitudes, as a measurable psychological construct, are indicative of enduring stability, and they are positively correlated with the occurrence of behavior. Therefore, by gaining a deeper understanding of residents' attitude formation, targeted educational and promotional activities can be designed to foster positive attitudes, ultimately driving concrete conservation behaviors.

Furthermore, residents' attitudes play a mediating role between economic value, aesthetic value, historical value, social value, perceived behavioral control, and residents' behavioral intentions. These results align with the earlier studies [3,42,44,60,61]. This indicates that the association of the independent variables on the dependent variable is not direct but is transmitted through the intermediate link of residents' attitudes. By strategically reinforcing and promoting multidimensional value perceptions such as economic value, aesthetic value, historical value, and social value, it is possible to enhance residents' cultural conservation attitudes more effectively. Additionally, considering factors related to perceived behavioral control in intervention measures, enhancing residents' perceived control over cultural conservation behaviors will also have a positive impact on the formation of behavioral intentions. In summary, the discovery of mediating effects enriches our understanding of the formation of attitudes toward protecting ICH, providing theoretical support for more specific and targeted cultural conservation measures.

### 5.1 Theoretical and practical contributions

#### 5.1.1 Theoretical contributions

Theoretically, this study examines the relationship between VAB model and TPB theory for Gejia residents' protection intentions. This study confirms that economic value, social value, historical value, aesthetic value and perceived behavioral control have positive and significant relationships with residents' behavioral intentions. By integrating these two models, we can provide deeper insights into the complex factors influencing residents' protective behavioral intentions and contribute to information system literature.

Furthermore, the theoretical significance of this study also lies in its pioneering integration of the revised VAB model and the TPB model, providing an in-depth exploration of residents' willingness to protect Batik. By combining these two models, researchers can better understand the factors influencing individual decision-making. This integration enhances the model's alignment with real-world scenarios, thereby improving the scientific validity and reliability of the theoretical framework.

Finally, by studying residents' willingness to protect Batik, we can conduct an in-depth analysis of this specific ICH and provide theoretical guidance for protecting other ICH. This offers an innovative methodological framework for future research, facilitating a more comprehensive exploration and understanding of the social dynamics and behavioral patterns associated with protecting ICH. This holds significant implications for advancing academic research and practices in related fields.

#### 5.1.2 Practical contributions

The results of this study have particular practical significance for the government, community, inheritors, and other relevant stakeholders.

Firstly, this research finds that economic value, social value, historical value, aesthetic value have positive and significant relationships with residents' behavioral intentions. These research results can guide local government in taking actions. For example, the government can stimulate residents' interest and love for batik and enhance their protection intention by improving their sense of identity for the historical value, social value, economic value, and aesthetic value of Gejia batik.

Secondly, this study found that perceived behavioral control has the greatest impact on Gejia residents' intention to protect batik, implying that relevant stakeholders, such as the academic community and research institutions, can deeply study the mechanism of influence of perceived behavioral control on cultural inheritance and protection. Thus, providing more scientific theoretical support and practical experience for cultural heritage protection. In addition, this research result also indicates that batik inheritors can encourage local residents' participation in batik protection through providing technical training and support.

Finally, attitude plays a mediator role among economic value, aesthetic value, social value, historical value, and perceived behavioral control with behavioral intention. These results indicate that in the practice of promoting residents’ willingness to protect batik, it is important to focus on Gejia residents' behavioral attitudes. By strengthening positive awareness and shaping attitudes towards batik, the local community can enhance residents' intentions to protect it and promote the inheritance and development of batik.

### 5.2 Limitations and future research

As with other studies, although most of the hypotheses in this study are supported, there are still some limitations in research design and methodology. First, the survey selected the four villages that were the gathering place of the Gejia people, which did not cover the whole Gejia population, and the conclusion reached could not represent the whole Gejia population. Therefore, future studies can investigate a more extensive scope so that the conclusions are universal. Second, the purposive sampling method, a non-probability sampling method, is adopted in this work in which researchers select the respondents purposefully according to the needs of research purposes and research problems. The representativeness of samples is poor, and the conclusions cannot be easily generalized to the whole group. Therefore, in future research, we can adopt the random sampling method so that every individual has a chance to be selected as a part of the sample, and the popularization of the research conclusion is more reliable. Third, this study only considered critical variables from the VAB and TPB models to explain residents' behavioral intention to protect Batik. All independent variables explained only 46.3 % of the variance in behavioral intention. Statistically, the explanatory power of these independent variables on the dependent variable only reached a moderate level. This suggests that there are potentially more critical variables influencing Gejia residents' behavioral intentions. Therefore, in future research, consideration could be given to incorporating additional variables, such as cultural identity, perceived benefits, and so on, to enhance the explanatory power of the independent variables. Fourth, This study is a cross-sectional study, with data collection concentrated at a specific time point. Therefore, it is unable to determine causal relationships between variables or track changes in variables over time. Therefore, future research can conduct surveys on Gejia residents through longitudinal research to explore causal relationships between variables and changes and trends in the attitudes and behaviors of Gejia residents. Lastly, the ultimate focus of this study is residents' behavioral intention to protect batik, and there is often a gap between behavioral intention and actual behavior. Behavioral intention does not always accurately predict actual behavior as it may influenced by other factors. Therefore, in future research, including actual behavior in the research framework would be beneficial.

## Ethics statement

This study was conducted after receiving approval and permission from the Jawatankuasa Etika Penyelidikan Manusia Universiti Sains Malaysia (code: USM/JEPeM/PP/23020174). Because the data for this study were mainly collected through a questionnaire, it was not possible for everyone to fill in an informed-consent form, such as when an interview is used. Therefore, to simplify the process, we set an informed-consent option at the beginning of the questionnaire, and those who checked this option indicated that they granted informed consent. Moreover, we confirmed that informed consent was obtained from all the participants in our survey.

## Funding

This research was funded by the General Project of Philosophy and Social Sciences for Universities in Jiangsu Province, grant number (2022SJYB2012).

## Data availability statement

Due to ethical-review requirements, research-related data are not kept in publicly available repositories, and all research data is accessible only to researchers. Anyone who needs data to reproduce research results can email the author to obtain the data.

## CRediT authorship contribution statement

**Xizhen Li:** Writing – review & editing, Writing – original draft, Visualization, Validation, Supervision, Software, Resources, Project administration, Methodology, Investigation, Funding acquisition, Formal analysis, Data curation, Conceptualization. **Nurul Hanim Romainoor:** Writing – review & editing, Supervision.

## Declaration of competing interest

The authors declare that they have no known competing financial interests or personal relationships that could have appeared to influence the work reported in this paper.

## Figures and Tables

**Table A:** Independent-sample *t*-test results for each item

Item	T-test for Equality of Means					N	Mean	Std. Deviation
	t	df	Sig. (2-tailed)	Mean Difference	Group			
AV1	−10.749	50	0.000	−2.281	1.00	20	4.25	1.020
					2.00	32	6.53	0.507
AV2	−9.772	50	0.000	−2.269	1.00	20	3.20	0.951
					2.00	32	5.47	0.718
AV3	−10.926	50	0.000	−2.438	1.00	20	3.75	1.020
					2.00	32	6.19	0.592
AV4	−1.874	50	0.067	−0.263	1.00	20	5.30	0.470
					2.00	32	5.56	0.504
EV1	−8.459	49	0.000	−1.792	1.00	25	4.40	0.866
					2.00	26	6.19	0.634
EV2	−10.377	49	0.000	−2.306	1.00	25	4.04	0.935
					2.00	26	6.35	0.629
EV3	−10.134	49	0.000	−2.169	1.00	25	3.60	0.816
					2.00	26	5.77	0.710
EV4	−10.237	49	0.000	−2.454	1.00	25	4.20	1.118
					2.00	26	6.65	0.485
HV1	−8.966	40	0.000	−1.982	1.00	22	4.82	0.907
					2.00	20	6.80	0.410
HV2	−9.730	40	0.000	−2.273	1.00	22	4.23	0.813
					2.00	20	6.50	0.688
HV3	−11.248	40	0.000	−2.455	1.00	22	4.05	0.844
					2.00	20	6.50	0.513
HV4	−8.464	40	0.000	−1.782	1.00	22	4.32	0.716
					2.00	20	6.10	0.641
HV5	−9.454	40	0.000	−2.295	1.00	22	3.95	0.844
					2.00	20	6.25	0.716
SV1	−9.868	38	0.000	−2.350	1.00	20	3.50	0.761
					2.00	20	5.85	0.745
SV2	−12.945	38	0.000	−2.750	1.00	20	3.30	0.571
					2.00	20	6.05	0.759
SV3	−9.400	38	0.000	−2.350	1.00	20	3.80	0.768
					2.00	20	6.15	0.813
SV4	−1.094	38	0.281	−0.200	1.00	20	3.75	0.444
					2.00	20	3.95	0.686
SN1	−11.735	50	0.000	−1.894	1.00	22	3.27	0.703
					2.00	30	5.17	0.461
SN2	−12.627	50	0.000	−2.385	1.00	22	3.18	0.733
					2.00	30	5.57	0.626
SN3	−10.629	50	0.000	−2.227	1.00	22	3.27	0.767
					2.00	30	5.50	0.731
SN4	−1.451	50	0.153	−0.258	1.00	22	2.41	0.590
					2.00	30	2.67	0.661
PBC1	−11.046	42	0.000	−2.081	1.00	21	3.57	0.746
					2.00	23	5.65	0.487
PBC2	−11.540	42	0.000	−2.054	1.00	21	3.38	0.669
					2.00	23	5.43	0.507
PBC3	−10.168	42	0.000	−2.023	1.00	21	3.24	0.831
					2.00	23	5.26	0.449
PBC4	−10.385	42	0.000	−2.110	1.00	21	3.24	0.831
					2.00	23	5.35	0.487
ATT1	−17.978	38	0.000	−2.850	1.00	20	3.25	0.550
					2.00	20	6.10	0.447
ATT2	−13.215	38	0.000	−2.900	1.00	20	3.85	0.875
					2.00	20	6.75	0.444
ATT3	−15.286	38	0.000	−3.400	1.00	20	3.50	0.946
					2.00	20	6.90	0.308
ATT4	−17.166	38	0.000	−3.450	1.00	20	3.15	0.745
					2.00	20	6.60	0.503
BI1	−18.653	38	0.000	−3.080	1.00	19	3.16	0.501
					2.00	21	6.24	0.539
BI2	−16.744	38	0.000	−3.323	1.00	19	3.11	0.737
					2.00	21	6.43	0.507
BI3	−17.448	38	0.000	−3.632	1.00	19	3.37	0.955
					2.00	21	7.00	0.000
BI4	−20.006	38	0.000	−3.880	1.00	19	2.26	0.653
					2.00	21	6.14	0.573
