# Supplementary material for: Explaining residents' support to protect Gejia batik through the value-attitude-behavior model and theory of planned behavior
Source: Heliyon. 2024 Apr 25;10(9):e30205. doi: 10.1016/j.heliyon.2024.e30205 (PMC11078639; doi:10.1016/j.heliyon.2024.e30205)

**Explaining residents' support to protect Gejia Batik**

Dear Participant,

Thank you for taking the time to participate in this research study. Your input is valuable and will contribute to the advancement of knowledge in protecting Intangible cultural heritage.

Before you proceed with the questionnaire, we kindly ask you to read the following information carefully.

**Informed Consent for Participation in Survey**

**Title**: [Explaining residents' support to protect Gejia Batik]

**Researcher:** [LI XIZHEN]

**Contact Information:** [379076203@qq.com / 15161986239]

**Introduction:**

You are invited to participate in a research survey conducted by Li Xizhen, as part of a study on explaining residents' support to protect Gejia batik. Your voluntary participation in this survey is greatly appreciated. Before you decide to participate, we kindly ask you to read this consent form and understand the information provided. If you have any questions or concerns, please feel free to contact us using the provided contact information.

**Purpose of the Study:**

The purpose of this survey is to explain residents' support for protecting Gejia Batik in southeast Guizhou. By participating in this survey, you will help us gain valuable insights into factors that influence residents' attitudes and willingness to protect Gejia batik.

**Procedures:**

If you agree to participate in this survey, you will be asked to answer a series of questions related to your value cognition, attitude, and behavior intention toward Gejia Batik. The estimated time to complete the survey is approximately 5-10 minutes.

**Voluntary Participation:**

Your participation in this survey is entirely voluntary. You have the right to refuse to participate or withdraw your participation at any time without facing any negative consequences. Your decision to participate or not will not affect your relationship with us.

**Confidentiality and Anonymity:**

Your responses will be kept confidential to the extent permitted by law. Any information that is obtained during the survey will be anonymized, and no personally identifiable information will be linked to your responses. Only the researchers involved in this study will have access to the data, and all data will be stored securely.

**Risks and Benefits:**

There are no foreseeable risks associated with participating in this survey. Your participation will be beneficial in providing valuable insights that may contribute to the protection of Gejia Batik.

**Data Usage:**

The data collected in this survey will be used for research purposes only. The findings may be used in research reports or publications; however, your responses will remain anonymous.

**Contact Information:**

If you have any questions or concerns about this survey or the research in general, please feel free to contact [Li Xizhen] at [379076203@qq.com / 15161986239].

By proceeding with this survey, you indicate that you are at least 18 years old and have read and understood the information provided in this consent form. Your decision to participate is voluntary, and you may withdraw your consent at any time during the survey.

A √ in the box where I agree indicates that you have read and understood the information provided in this consent form and voluntarily agree to participate in the survey.

- **I agree to participate in the survey.**
- **I do not agree to participate in the survey.**

If you volunteer for this study, you can start answering.

Date: 15/08/2023

**Section A Demographic Profile**

Please tick （√） at the relevant box.

1. Sex: Male Female
2. Ethnic group: Han Gejia Others
3. Age: 18-29 30-39

40-49 50-60

1. what is your marital status?

Married

Unmarried

1. What is your educational level?

High school or below

Collge

University

Postgraduate

1. What's your monthly income now?

<1000￥ 1000-3000￥ 3001-5000￥

5001-8000￥ 8001-10000 ￥ >10000￥

1. Which village are you from?

Fengxiang village Wangba village Tangdu village

Matang village

1. How many years have you lived in this village?

< 10 years

10-20 years

21-30years

>30years

**Section B Choosing your innermost thoughts according to the description**

(Please tick [√] in the relevant box)

1. I think the commercial development of Gejia Batik can benefit the inheritor.
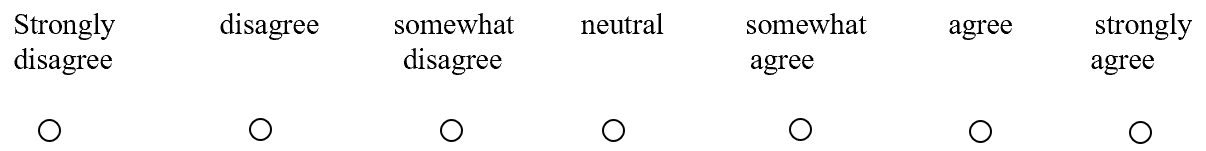

2. Gejia Batik is a reflection of and witness to historical development.


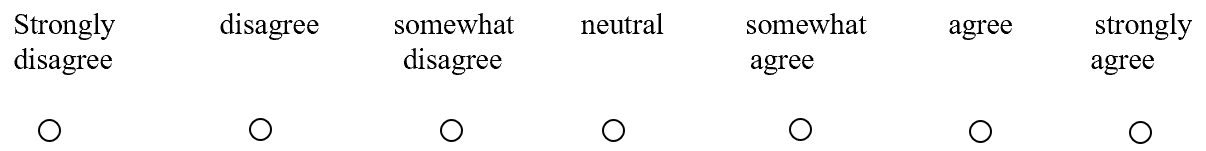


1. I think Gejia Batik aligns with contemporary people's aesthetics.

 
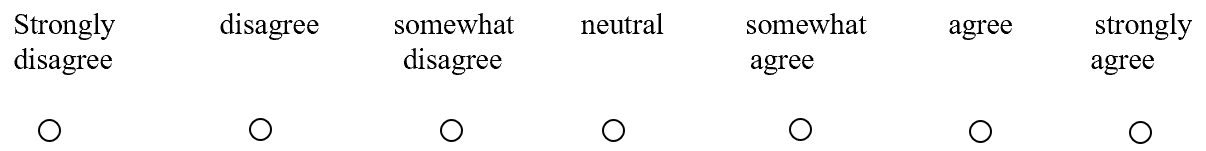


1. I think Gejia Batik reflects cultural diversity.


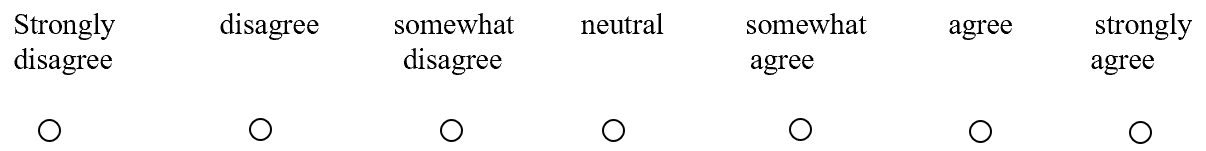


1. I can perform work to support the development of Gejia Batik.


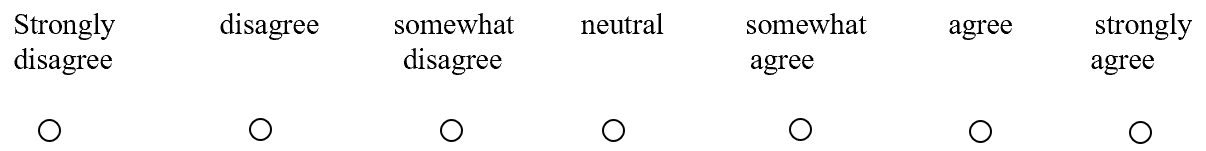


1. People whose opinions I value would prefer that I support Gejia Batik’s development.


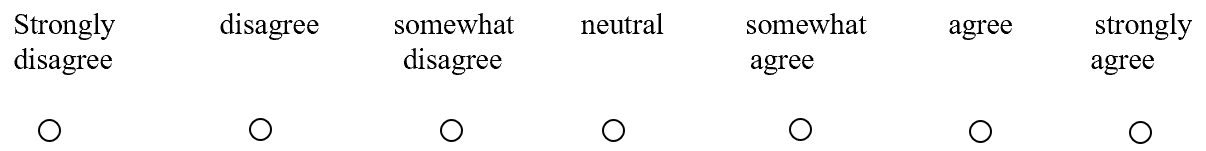


1. I think Gejia Batik has a cultural influence.


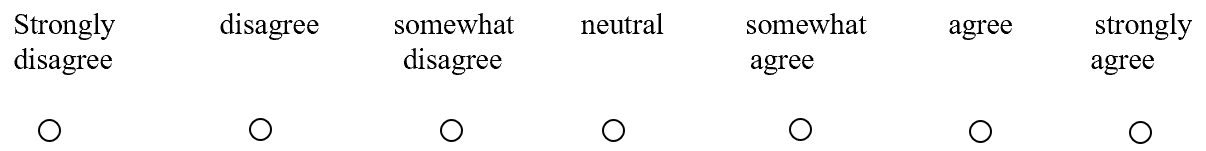


1. Gejia Batik has a long history.


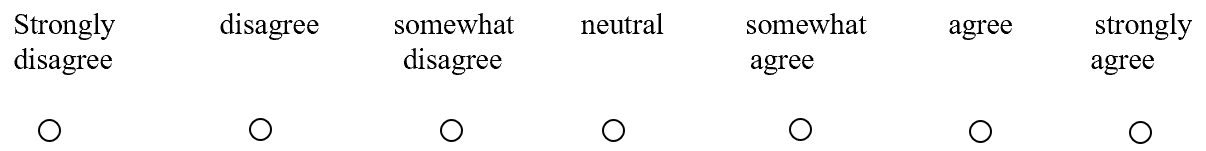


1. I think developing Gejia Batik can generate economic income for the local area.


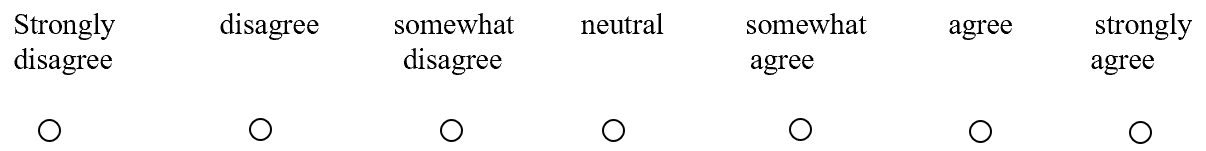


1. Gejia Batik is a reflection of the cultural tradition of the community.


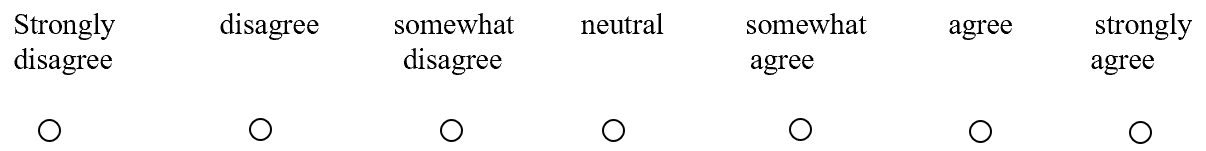


1. I think Gejia Batik has economic value.


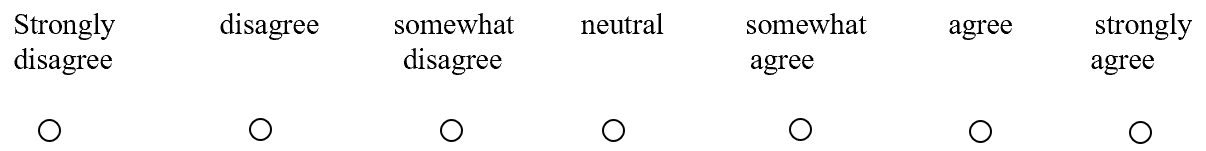


1. Gejia Batik continues history and culture in the contemporary era.


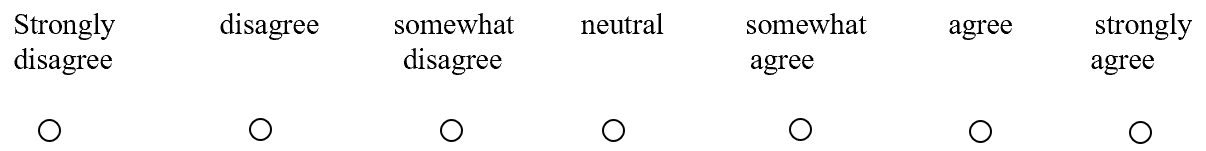


1. People who are important to me think I should support Gejia Batik’s development.


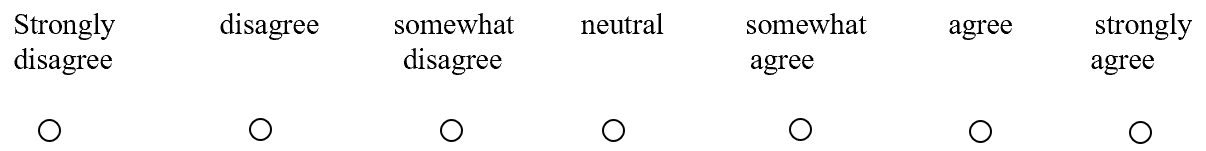


1. I have the skills to perform work to support the development of Gejia Batik.


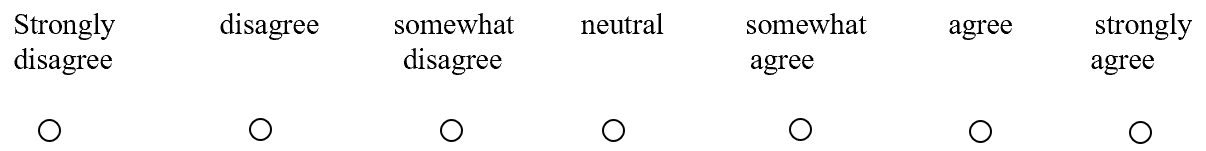


1. People who are vital to me suggested I protect Gejia Batik.


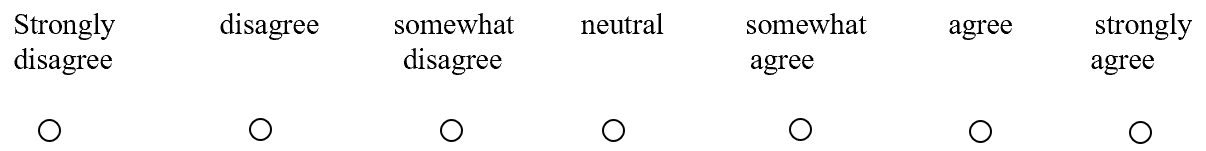


1. I think Gejia Batik has artistic appeal.


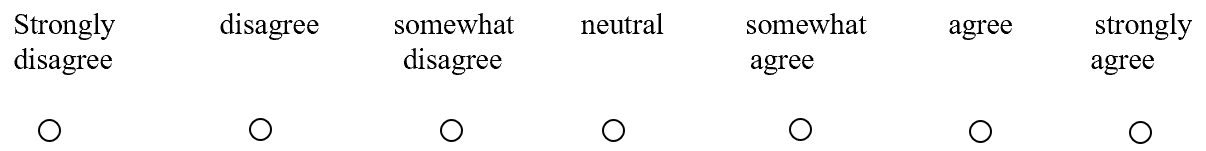


1. I have the talent to perform work to support the development of Gejia Batik.


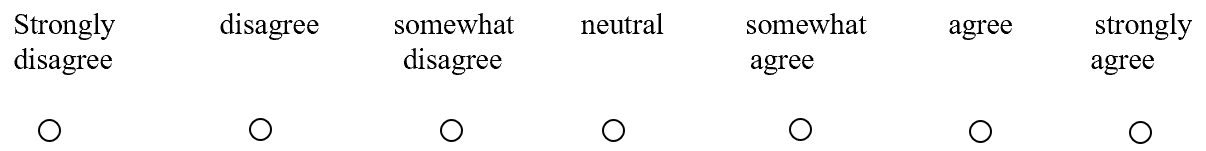


1. I think Gejia Batik can give people aesthetic enjoyment.


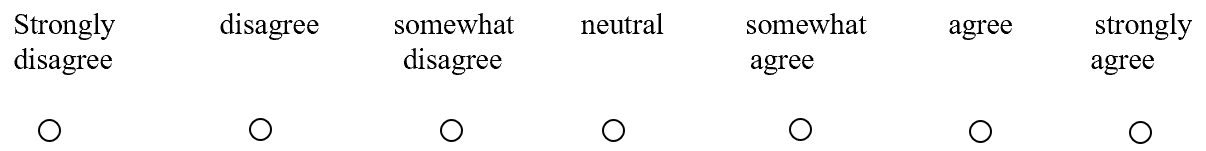


1. I am very interested in protecting Gejia Batik.


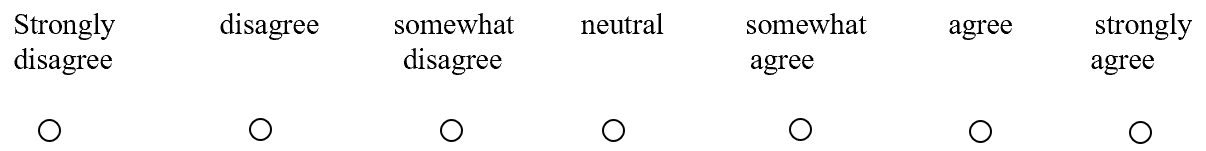


1. I am willing to support the development of Gejia Batik.


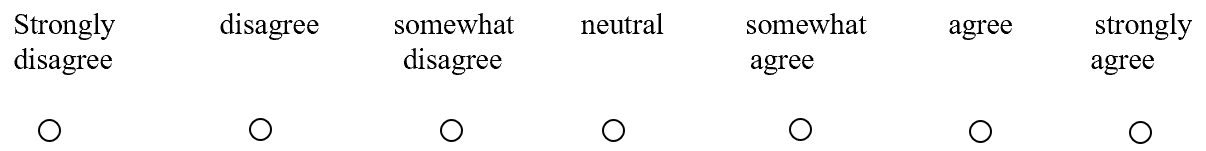


1. Gejia Batik supplements the deficiency of traditional historical records.


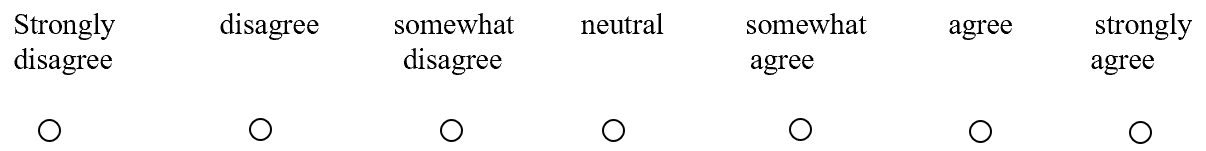


1. I think Gejia Batik is worth protecting.


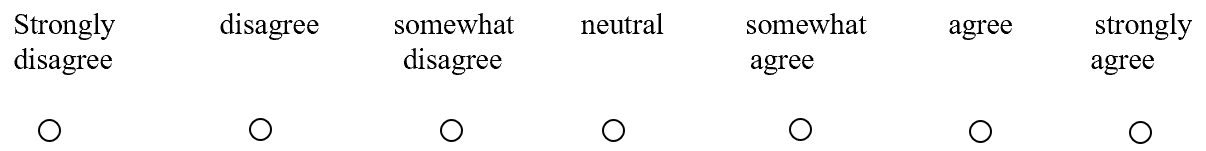


1. I will make an effort to support the development of Gejia Batik.


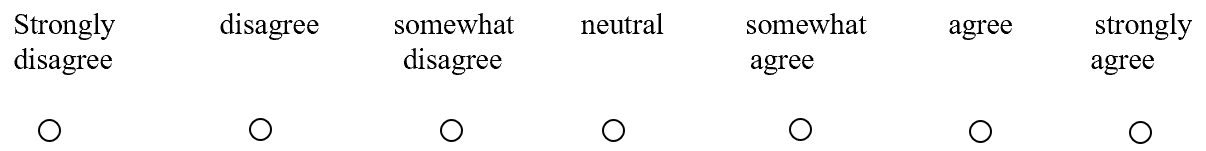


1. I have time to support Gejia Batik’s development.


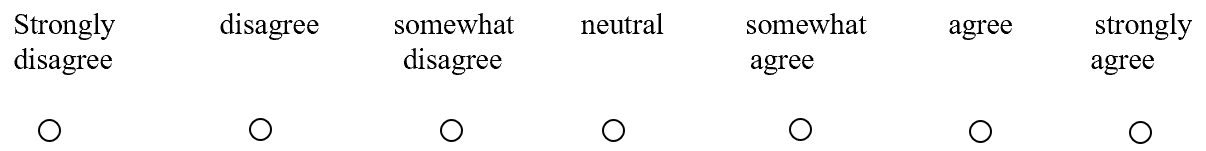


1. I plan to support the development of Gejia Batik


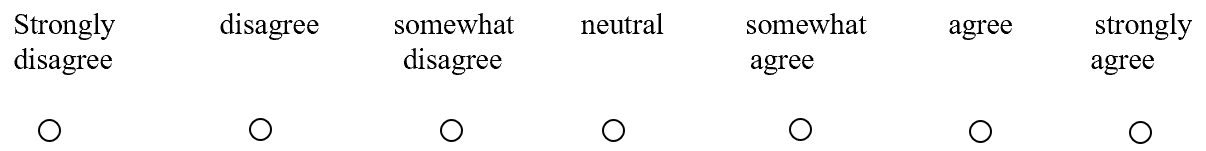


1. I think Gejia Batik is an essential achievement of human civilization.


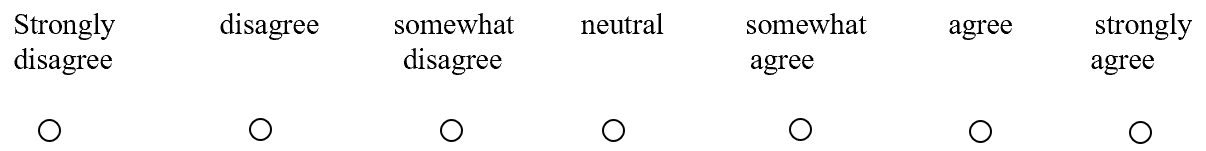


1. I have the responsibility to protect Gejia batik.


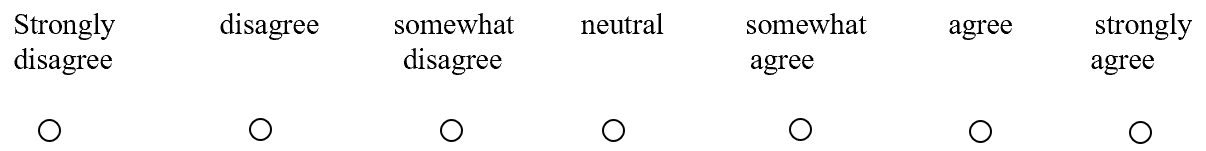


1. I want to work in the conservation of Gejia batik.


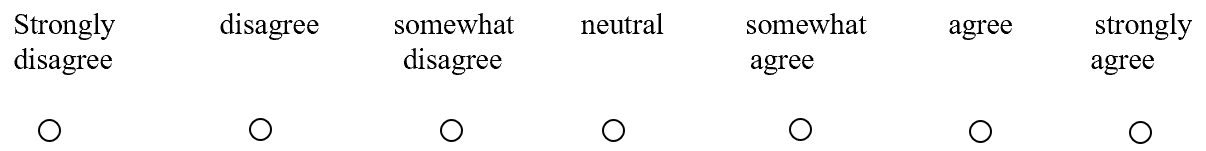


1. I think it is essential to protect Gejia Batik


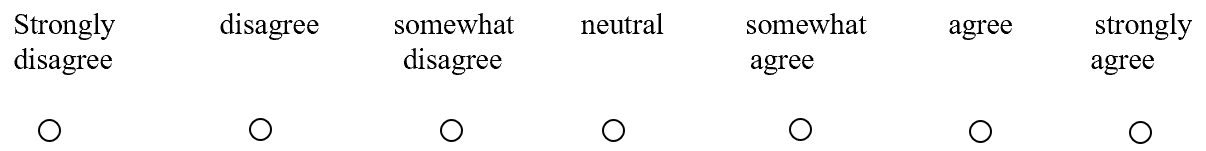


1. I think the protection of Gejia Batik can promote the employment of local people.


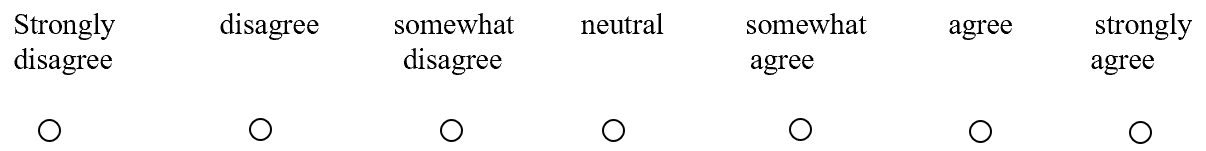

Supplement: Multimedia component 1 [file mmc1.docx]
